# Visual analytics of geo-social interaction patterns for epidemic control

**DOI:** 10.1186/s12942-016-0059-3

**Published:** 2016-08-10

**Authors:** Wei Luo

**Affiliations:** Geography Department, University of California, Santa Barbara, Santa Barbara, CA USA

**Keywords:** Geo-social interaction patterns, Varying spatial–social scales, Geo-social visual analytics, Agent-based epidemic models, Social network analysis, Epidemic control

## Abstract

**Background:**

Human interaction and population mobility determine the spatio-temporal course of the spread of an airborne disease. This research views such spreads as geo-social interaction problems, because population mobility connects different groups of people over geographical locations via which the viruses transmit. Previous research argued that geo-social interaction patterns identified from population movement data can provide great potential in designing effective pandemic mitigation. However, little work has been done to examine the effectiveness of designing control strategies taking into account geo-social interaction patterns.

**Methods:**

To address this gap, this research proposes a new framework for effective disease control; specifically this framework proposes that disease control strategies should start from identifying geo-social interaction patterns, designing effective control measures accordingly, and evaluating the efficacy of different control measures. This framework is used to structure design of a new visual analytic tool that consists of three components: a reorderable matrix for geo-social mixing patterns, agent-based epidemic models, and combined visualization methods.

**Results:**

With real world human interaction data in a French primary school as a proof of concept, this research compares the efficacy of vaccination strategies between the spatial–social interaction patterns and the whole areas. The simulation results show that locally targeted vaccination has the potential to keep infection to a small number and prevent spread to other regions. At some small probability, the local control strategies will fail; in these cases other control strategies will be needed. This research further explores the impact of varying spatial–social scales on the success of local vaccination strategies. The results show that a proper spatial–social scale can help achieve the best control efficacy with a limited number of vaccines.

**Conclusions:**

The case study shows how GS-EpiViz does support the design and testing of advanced control scenarios in airborne disease (e.g., influenza). The geo-social patterns identified through exploring human interaction data can help target critical individuals, locations, and clusters of locations for disease control purposes. The varying spatial–social scales can help geographically and socially prioritize limited resources (e.g., vaccines).

## Background

Airborne infectious diseases (e.g., influenza) cause a huge cost to society. The 1918 influenza pandemic infected one-third of the world’s population and caused 50 million deaths worldwide [1]. Severe acute respiratory syndrome (SARS) and Swine/H1N1 Influenza had a dramatic impact over most of the world in the twenty-first century [2, 3]. Although the world’s public health system has made tremendous efforts to detect, prepare, and control such epidemics, outbreaks of novel infections (e.g., the Middle East respiratory syndrome) continue to occur, exacerbated by the increasing urbanization and the mobility of contemporary society [4]. How to effectively control airborne infectious disease transmission is still an open question.

This research views the spread of airborne diseases as geo-social interaction problems, because human interaction connects different groups of people over geographical locations where the viruses transmit [5]. Research has demonstrated that a better understanding of the underlying network structure of a population at risk to infectious disease gives insight into disease dynamics and control strategies [6–9]. Guo [10] discovered geo-social interaction patterns in population mobility data which provide great potential in designing effective pandemic mitigation. However, little work has been done to evaluate the effectiveness of control strategy design using such geo-social interaction patterns [11]. Thus, this research proposes a new framework in terms of an effective disease control strategy that should start with identifying geo-social interaction patterns, progresses to designing effective control measures according to those patterns, and ends with control measure evaluation. This research designs and develops a visual analytics tool using the framework described.

The visual analytics tool aims to achieve the following linked research objectives: (1) to develop visual analytics methods representing complex human interaction data as geo-social forms that can facilitate the discovery of patterns in terms of disease spread and transmission control; (2) to develop methods to transform discovered patterns into reliable knowledge to support decision-making processes in epidemic control. The tool consists of three components: a reorderable matrix for geo-social mixing patterns, agent-based epidemic models, and combined visualization methods. The reorderable matrix allows users to identify useful geo-social interaction patterns in terms of disease transmission and control. The combined visual-computational methods allow users to transform the useful patterns into knowledge to design advanced control scenarios. The agent-based epidemic models allow users to evaluate the efficacy of the control scenarios when considering such geo-social interaction patterns.

## Related research

In terms of the epidemic control, this study addresses decisions about vaccination, and implements various immunization strategies into the geo-social visual analytics tool to allow the design and testing of advanced control scenarios. A comprehensive review about all of the control strategies used in the previous research can be found in Lee et al. [12]. There is also research that integrates population-based epidemic models into visual analytics [13, 14].

### Agent-based epidemic models and vaccination strategies

Contact networks are built by a series of individuals with social or spatial locations and links between those individuals, which are fundamentally linked to the spatial spread of infectious disease [15]. Agent-based epidemic models are based on those contact networks, in which each individual is regarded as an agent and links between individuals represent possible infection channels [16, 17]. Each agent in the population is assigned to an infection status (e.g., susceptible, infectious, or recovered). Infection dissemination over those contact networks depends on the likelihood of infection and individual-level human interactions [18]. At this point, network topology plays a significant role in the speed and extent of epidemic dynamics within a population [19]. Therefore, epidemiologists currently use agent-based epidemic models to simulate disease transmission and corresponding control scenarios on different network structures.

Given the limited supply of vaccines, vaccinations aim to achieve the highest efficacy through immunizing a fraction of the population [20, 21]. Current vaccination strategies identify the targeted population with the combinations of different network relationships among individuals [7, 22–25], and can be called as contact-based strategies. Some research has suggested that individuals who are socially close to an infection should have a high priority to be vaccinated, such as family members or office mates of those individuals [26, 27]. Other research has reported that targeting individuals with a large number of social contacts for immunization is an effective control strategy [24, 28], when community structure is not strong. When community structure becomes stronger, targeting individuals bridging communities becomes more effective than targeting individuals with a large number of social contacts [29, 30]. Those studies have shown that disease transmissions can be controlled through immunizing a small number of the “proper” individuals in a social network [18, 20, 31].

The above control strategies only focused on social contact characteristics without considering human spatial interaction patterns that play a vital role in shaping disease spread process. The typical intervention strategy relevant to spatial distance is to simply apply a certain distance threshold (e.g., 5 km) to prohibit long-range trips’ transmission [32]. However, human interaction patterns are much more complicated than a simple distance threshold, given that population mobility connects different groups of people over geographical locations with varying distances. Previous research shows that disease transmission (i.e., influenza) starts with a local growth followed by a long distance transmission to the whole population [33]. It indicates that there are three important characteristics in terms of disease transmissions: the locations of infection sources, human interactions within locations, and the movement of individuals among different locations. The first two characteristics determine the early stage of disease transmission patterns. The last characteristic describes the time course and geographic spread of the disease outbreak at the subsequent stage. The three characteristics determine human geo-social interaction patterns, based upon which researchers can design effective control strategies before airborne diseases occur. Thus, this research proposes that a new framework in terms of an effective disease control should identify geo-social interaction patterns first and then transform human interaction patterns into knowledge to design and evaluate the efficacy of control scenarios.

### Visual analytics in agent-based epidemic models

Visual analytics aims to leverage the power of human reasoning and computational analysis through visual interfaces that enable analysts with domain knowledge to turn complex data into useful information and knowledge, and to support real-world decision-making [34]. Several studies have been done to integrate agent-based epidemic models with visual analytics tools, in order to allow users to enable analysts to set up parameters to simulate disease transmission and design control scenarios. The Epi-Fast tool allows for a disease transmission and public health intervention simulation based on the explicit representation of social contact networks among individuals [35, 36]. The epidemic models and interventions are pre-configured into the tool, so it does not allow users to explore the social contact networks to identify human interaction patterns to design advanced control scenarios. Guo [10] develops a visual analytics tool to allow the identification of human interaction patterns, but it does not support designing and evaluating the efficacy of control scenarios considering the patterns.

The above discussion illustrates the new framework for an effective disease control processes. However, little work in visual analytics research areas has been done to evaluate the efficacy of control scenarios when considering geo-social interaction patterns. Thus, this research aims to implement the new framework through a visual analytics framework in order to fill the gap for both design of control strategies and visual analytics.

## Methods

### Data

This study uses data on face-to-face interactions among 242 individuals including 232 children and ten teachers, across ten classes over 2 days (Thursday, October 1st 2009 and Friday, October 2nd 2009) in a French primary school collected by Stehle et al. [37] (the data set is available at http://www.sociopatterns.org/datasets/primary-school-cumulative-networks/). Stehle et al. [37] used a proximity-sensing infrastructure based on radiofrequency identification devices (RFID) [38] to detect high-resolution proximity (about 1–1.5 m) and captured 77,602 contact events between individuals at the primary school, in order to capture close proximity interactions (CPIs) for the study of infectious disease transmissions. Each node represents individuals and edges are face-to-face interactions without directions. Each node has the attribute of *classname* that indicates the corresponding grade level and class number, and the teachers are assigned as the class of “Teachers” (Table 1). Edges between two nodes use the attribute “duration” to describe the cumulative time between two nodes in face-to-face proximity within 1 day (Table 2). The cumulative time is measured over an interval of 20 s that allows RFID to assess the proximity of two individuals with a probability over 99 % [38]. This study uses the data measured on the 2 days. This study converts the weighted edge table into the weighted networks among each individual. Each node has the attribute of *classname* to distinguish nodes from different communities. The data has been stored as comma-separated values (CSV) file format for input.

**Table 1:** Partial node table with two attributes: label indicates the id of each node, and classname indicates the corresponding class groups, including grade level and class number for students and “Teachers” for teachers

Label	Classname
1538	4A
1539	4A
1551	3B
1552	3B
1650	Teachers
1653	Teachers
1656	1B

**Table 2:** Partial edge table with four columns: Source indicates the id of source node, Target indicates the id of Target node, Type indicates that all edges are undirected, Duration indicates that the cumulative time between two nodes measured in seconds within 1 day

Source	Target	Type	Duration
1538	1539	Undirected	260
1538	1545	Undirected	120
1538	1546	Undirected	660
1538	1548	Undirected	60
1538	1549	Undirected	40
1538	1618	Undirected	360
1538	1653	Undirected	420

The high-resolution human interaction data is chosen for four reasons. First, the resolution of contact network data from other collections relevant for infectious disease transmission is too coarse for airborne disease transmission; these include surveys, socio-technological networks, mobile devices, and large scale human interaction simulation models [7]. Second, our research aims to evaluate the effectiveness of control strategy using human interaction patterns and the high-resolution data used in this research can capture the CPIs relevant to disease transmission. Third, schools are considered to play an important role in infectious disease spread such as influenza mainly because of the high density of CPIs [39] and the high-resolution data provides an opportunity to design micro-interventions considering human interaction patterns and compare the outcomes of alternative mitigation measures. Lastly, before the availability of high-resolution contact network data from school environments, school closure has been proposed as an effective mitigation strategy [40]. Such measures however cause high associated social and economic costs. With the high-resolution contact network data, Gemmetto et al. [39] have suggested that targeted grade closure strategies can achieve results that are almost as effective as the school closure, at a much lower cost. The targeted grade closure strategies focus more on social aspects of network structures (i.e., the targeted class and its corresponding grade), but they do not take full advantage of the high-resolution data that can describe the real human spatial–social interaction patterns.

### Geo-social epidemic visual analytics (GS-EpiViz)

This study treats each class as the basic local human interaction unit for two reasons: the network density for within class interactions is significantly higher than between class interactions; it is practical to implement control strategies based on spatial confinement (e.g., class, household, school). Based on the basic unit, the average time duration, i.e., total time duration/(the basic time measure unit of 20 s × all potential connections), between two communities in the network is used to measure the social connection strength among communities. Communities with strong social connections imply highly spatial interactions caused by individual mobility via which infections can spread. Disease transmissions that start from the individual-level within each class followed by the class-level transmissions via individual mobility can generate spatial–social interaction patterns in a hierarchical structure.

**Fig. 1:**
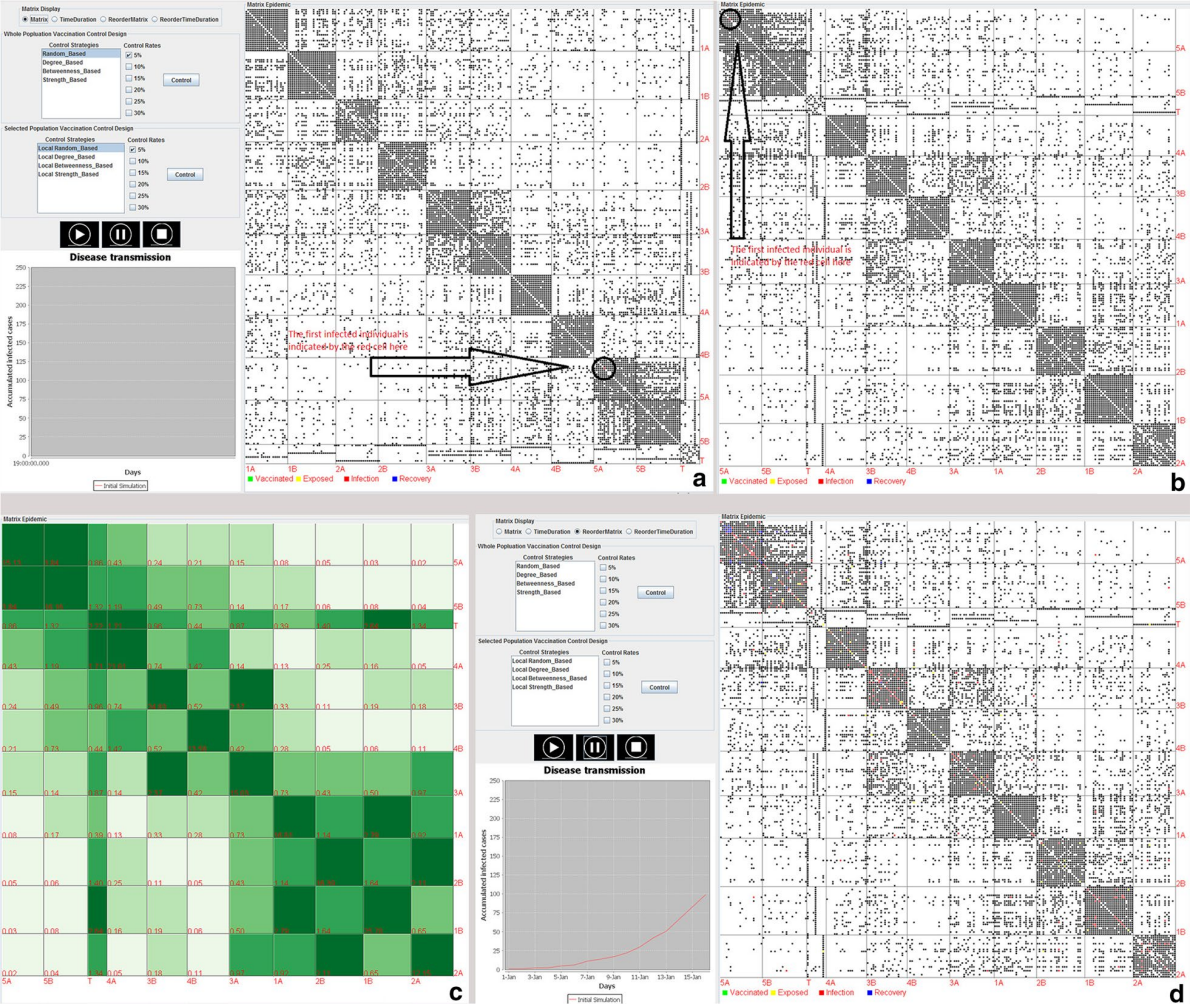
GS-EpiViz consists of four major components: display panel, control panel, xy plot, and matrix view. Display panel in the *top left* allows users to select different matrix displays: binary matrix, time duration matrix, reorder binary matrix, and reorder time duration matrix. Control panels allow users to design different control scenarios based on the whole population and selected population. XY plot displays the accumulated infected cases over time based on the simulation of agent-based epidemic models. The matrix view visualizes different matrix displays with class information on the *bottom* and on the *right*. *Black* cells in the matrix view represent human interactions at the individual level, and white cells indicate there are no human interactions. **a** Displays the binary network with *rows* and *columns* organized by grade and class from 1st grade class A at the *top left* to 5th grade class B in the *lower right*. **b** Displays the reordered binary network with new class numbers on the *bottom* and on the *right*. **c** Displays the reorder time duration network matrix with the average time duration values in each cell. **d** Displays a simulated influenza infection according to the parameters in Table 1 on the matrix view and XY plot. *Yellow* indicates that individuals are in an exposed state, *red* indicates an infectious state, and the *blue* indicates a recovery state

Visualizing the hierarchical structure of the social network in an appropriate way allows users to design vaccination strategies with regard to spatial–social interaction patterns. Network visualization has a rich history [41, 42] that has generated many variants on two primary categories of network visualization methods: node-link visualization and matrix-based visualization [43]. This study applies the matrix view to represent our network data (Fig. 1) for two reasons: a matrix view has the advantage to exhibit high-level structures (relationships between different communities) by finding the proper ordering of rows and columns [44]; the proper ordering of rows and columns in this application is determined by the communities in which infected cases are located. For example, the matrix view on the right in the Fig. 1a displays the interaction data with rows and columns organized by grade and class from 1st grade class A at the top left to 5th grade class B in the lower right. The first infected individual in the scenario is in grade/class 5A. Figure 1b reorders all classes in the matrix view: it puts group 5A, with the first infected case, on the top left followed by other groups according to the social connection strength from the highest to the lowest. The reordering process is of O(m + k^2^) complexity in which m represents the total number of edges and k represents the number of classes. The green cells along the diagonal from the top left to the bottom right in matrix views in Fig. 1c, d shows the within-community interactions, whereas all other cells show the spatial interactions among different communities. The interactive view allows users to dynamically adjust the reordering sequence according to the positions of infection sources and social connection strength between the classes with the infection sources and all other classes. In this way, users can identify the clusters of communities with the strongest spatial–social interactions and then focus vaccination or other preventative measures within those communities rather than applying the response uniformly to all communities. This targeted response is potentially more efficient (in overall use of resources) and more effective (in minimizing the proportion of individuals who are infected).

In addition to representing human interaction network data in the matrix view to support control strategy design, this tool also implements agent-based epidemic models from a scratch to simulate disease transmission and different control scenarios. Specifically, each individual in our data set is considered as an agent who is described as one of four disease states: susceptible, exposed, infectious, or recovered (SEIR) [45]; this is so-called SEIR agent-based modelling. A single influenza infection is randomly introduced into the network with all other initially susceptible individuals. Influenza is chosen as an exemplar because it is a common infectious disease with reasonably well known transmission characteristics. This study assumes that transmission can only occur during the day time, and only on weekdays (thus when the individuals involved are at the school). Though this simplifying assumption is not realistic, it allows an analyst to analyze the disease spread and design control scenarios starting from one single infected case without considering multiple introductions of infected cases.

After coming in contact with an infection, a susceptible individual has a transmission probability 0.0015 per 20 s of contact (basic duration measurement unit) to be infected. This value has been chosen because it generates values of *R*_0_ (the basic reproductive number) consistent with observed R_0_ of pandemic influenza (0.9–2.1) in previous studies [46, 47]. R_0_ is defined as the average number of secondary cases generated in a susceptible population [48]. The calculation of R_0_ in this case study follows the steps: randomly generate one new infected case 100 times, simulate disease transmissions 100 times for each new infected case within the network according to parameters from the Table 3, and then calculate average R_0_ for each simulation. Both of the derived R_0_ based on the two networks respectively are approximately equal to 1.8–1.9 which falls into the observed R_0_ pandemic influenza (0.9–2.1). Upon infection, the individual enters into the exposed period (infected but not infectious). The mean exposed days, 3 days, will be used in this simulation [49]. After the exposed period, an exposed individual will become symptomatic and infectious. The infectious period used in this project is 7 days, the mean days for patients who recovered [49]. This study assumes that individuals cannot be infected again after recovery. Figure 1d shows a simulated influenza infection according to the parameters in the Table 3. The tool can be applied to study other emerging infectious diseases (e.g., measles) directly with the input of their corresponding parameters.

**Table 3:** Basic simulation parameters for influenza diffusion

Parameters	Default value	Literature
Total humans in simulation	242	Stehle et al. [37]
Length of exposed period	3 days	Heymann [49]
Length of infectious period	7 days	Heymann [49]
R_0_	0.9–2.1	Mills et al. [46] and Ferguson et al. [47]
Infection probability	0.0015	Measured based on R_0_

**Fig. 2:**
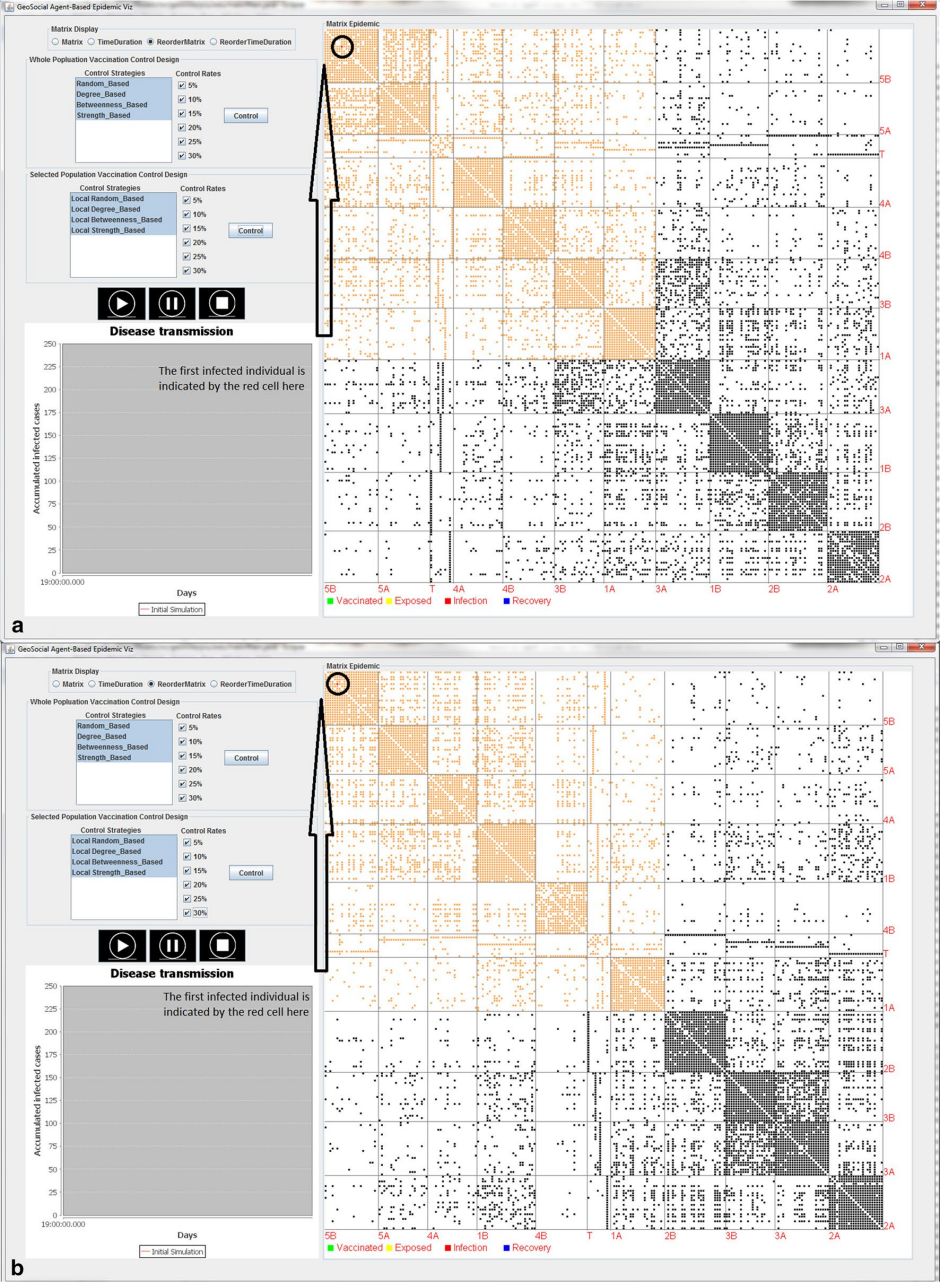
The design of eight vaccination strategies with six different control rates in the control panel, and selection of reorder binary matrix in the display panel. The matrix view visualizes the reordering network, Thursday, October 1st 2009 in **a** and Friday, October 2nd 2009 in **b**. The local control areas are highlighted in *orange*

In terms of control strategies, GS-EpiViz allows users to design four vaccination strategies: random-based, degree-based, betweenness-based, and strength-based vaccination strategies; these are selected because they are the most typical ones used to compare effectiveness of vaccination strategies based on different network structures [7, 22, 29]. The random-based vaccination strategy randomly identifies a fraction of the population to vaccinate. The basic idea of degree-based, betweenness-based, and strength-based strategies is first to rank the importance of individuals and then vaccinate the individuals from highest importance to lowest. The degree-based vaccination strategy ranks individuals according to the number of contacts during the day of measurement for vaccination. The betweenness-based vaccination strategy prioritizes individuals according to their betweenness centrality [50], capturing the extent to which a particular node lies on the bridge among different communities. The strength-based vaccination strategy ranks individuals according to their total time exposed to others during the day of measurement for vaccination. Given that the vaccination results are sensitive to vaccination rates, the tool provides a variety of options in terms of the percentage of the population vaccinated: 5, 10, 15, 20, 25, and 30 %. To compare the effectiveness of vaccination strategies based on the whole population and local human geo-social interactions, this tool provides two panels on the left of the tool: *Whole Population Control Design* and *Selected Population Control Design.* The former panel allows users to select control strategies and control rates based on the whole population, whereas the latter panel allows users to select control strategies and control rates based on the geo-social interaction patterns (orange area in the Fig. 2).

**Fig. 3:**
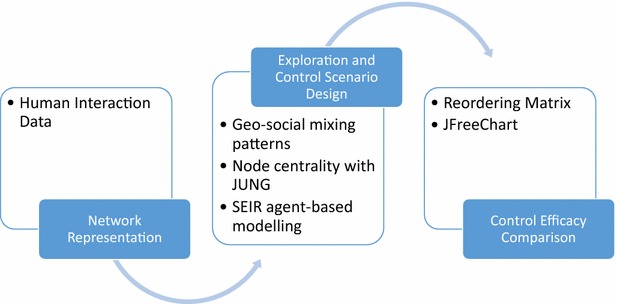
GS-EpiViz system architecture

Vaccination strategies are applied at the beginning of the spread of influenza in the network. The percent of the infected population and the spatial–social extent of infection are used to evaluate the efficacy of those strategies. There are two networks, four different control regions, four strategies, and six vaccination rates, yielding 192(2*4*4*4) combinations to simulate. The efficacy of vaccination strategies for each combination is estimated for 10,000 simulation runs, resulting in a total of 1,920,000 epidemic simulation runs. GS-EpiViz is developed with JAVA for a cross-platform purpose and the system architecture is illustrated in Fig. 3. Geo-social mixing patterns are identified from human interaction network data. Java universal network/graph framework (JUNG) is used to calculate node centrality (i.e., degree, betweenness) based on human interaction data. SEIR agent-based modelling is implemented to simulate disease transmission and control scenarios. Reordering matrix is implemented to identify and display geo-social mixing patterns of the data and simulation results from SEIR agent-based modelling. JFreeChart is used to display the vaccination efficacy results.

## Results

This study aims to compare the efficacy of vaccination strategies between the local geo-social interaction patterns and the whole areas based on the real world networks provided by the school interaction data. The better vaccination strategies are expected to generate a lower number of infections. Another measure of how well we can contain the epidemic locally is the number of infected cases occurring outside the selected areas. If this number is zero, the local control scenarios with the selected areas are considered successfully. Otherwise, the local control scenarios are considered as failures. Figure 2 shows the design of eight vaccination strategies with six different control rates in the two networks, Thursday, October 1st 2009 in (a) and Friday, October 2nd 2009 in (b). Two networks have been reordered according to the strength of social connections to the classroom with the first infected case. The local control areas are highlighted in orange. The efficacy of eight vaccination strategies in the two networks is evaluated through infection percentage and the spatial–social extent of infection as described below.

### Vaccination strategies in terms of infection percentage

**Fig. 4:**
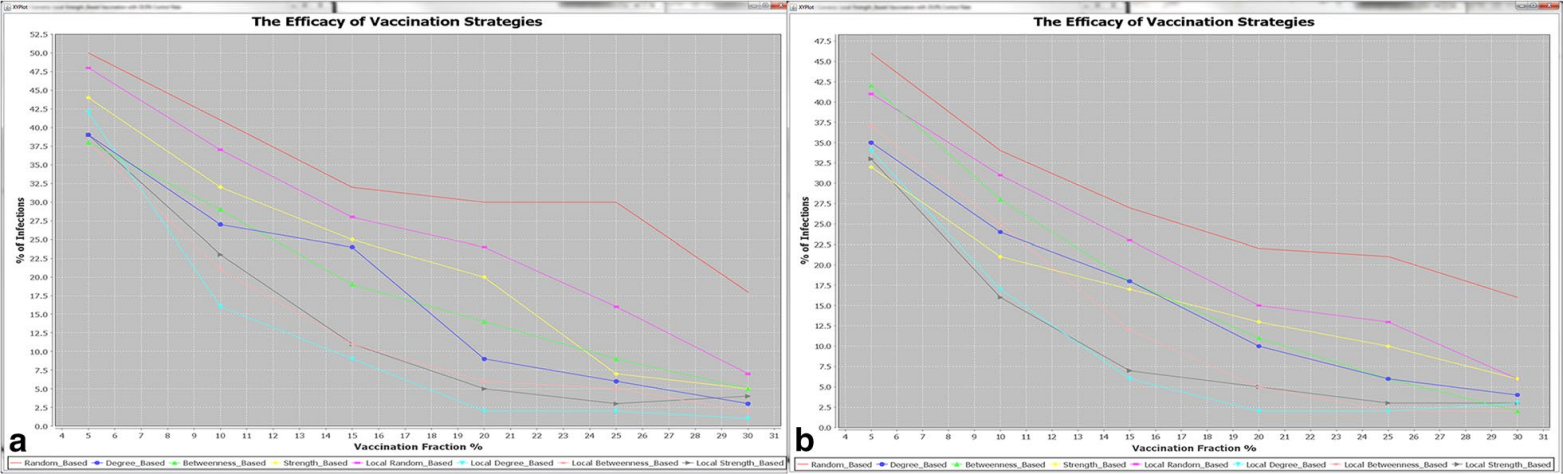
The efficacy of eight vaccination strategies in two networks, Thursday, October 1st 2009 in **a** and Friday, October 2nd 2009 in **b**. The *Y* axis represents the percent of infection, and the *X* axis represents the vaccination rates. Eight vaccination strategies are represented by eight curves in different *colors* and *shapes*, as the legend shows at the *bottom*

**Fig. 5:**
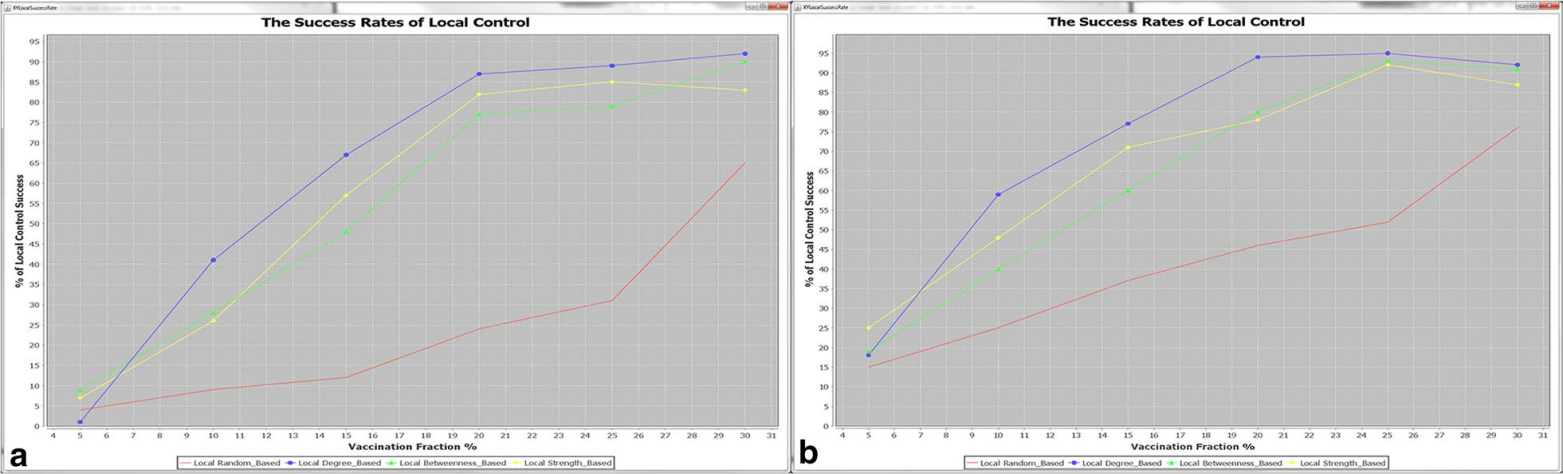
The percentage of the local control success for four local vaccination strategies within 10,000 simulation runs in two networks, Thursday, October 1st 2009 in **a** and Friday, October 2nd 2009 in **b**. The *Y* axis represents the percentage of the local containment success, and the *X* axis represents the vaccination fraction. Four local vaccination strategies are represented by four curves in different *colors* and *shapes*, as the legend shows at the *bottom*

**Fig. 6:**
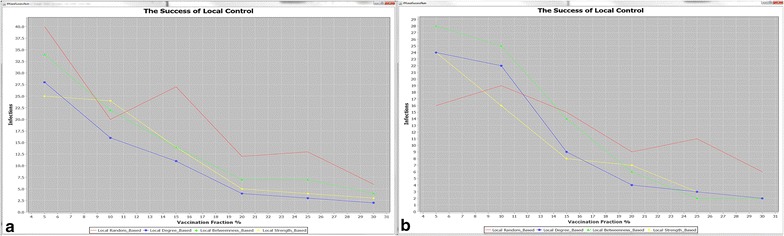
The total number of the final infected cases when the local vaccination strategies are successful within 10,000 simulation runs in two networks, Thursday, October 1st 2009 in **a** and Friday, October 2nd 2009 in **b**. The *Y* axis represents the total number of the final infected cases, and the *X* axis represents the vaccination fraction. Four local vaccination strategies are represented by four curves in different *colors* and *shapes*, as the legend shows at the *bottom*

**Fig. 7:**
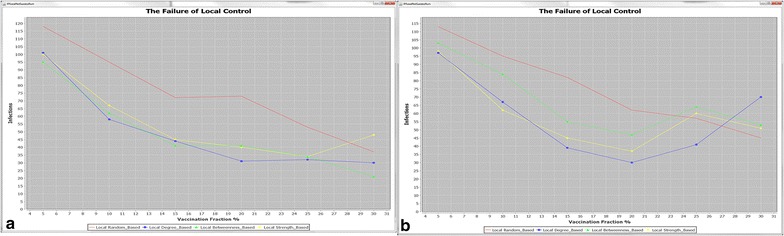
The total number of infections when the local vaccination strategies are not successful within 10,000 simulation runs in two networks, Thursday, October 1st 2009 in **a** and Friday, October 2nd 2009 in **b**. The *Y* axis represents the total number of the infected cases, and the *X* axis represents the vaccination fraction. Four local vaccination strategies are represented by four curves in different *colors* and *shapes*, as the legend shows at the *bottom*

The better vaccination strategies in terms of infection percentage are expected to generate a smaller number of infections. Figure 4a, b shows that all of the eight vaccination strategies can produce a decreasing number of infections in proportion to the increasing vaccination fractions. The random-based vaccination strategy produces the highest number of infections, followed by the random-based vaccination strategy with the local selected control areas in Fig. 2a, b. The other three pairs: degree-based, betweenness-based, and strength-based vaccination strategies exhibit the same pattern: the three strategies with the local selected control areas outperform those strategies with the whole area, respectively. The explanation for the pattern is illustrated in Figs. 5 and 6. Figure 5 displays the percentage of the local control success for the four local vaccination strategies within 10,000 simulation runs. As the vaccination fractions increase, all of the four local vaccination strategies can produce a higher percentage of the local control success, but to different degrees. In addition, when the local control scenarios are successful, they can produce a significantly lower number of infected cases (Fig. 6). Those results show that when the vaccination fraction reaches a relatively high level (i.e., 30 %) with the selected control areas, the disease transmission can be confined locally at a very high percentage (i.e., 90–95 %), resulting in only a small number of infections (i.e., 2–5). Figures 5 and 6 shows that the former can stop the disease transmission locally at a certain probability (Fig. 5), which also results in the lower number of infections (Fig. 6). Figure 4 shows that the disease transmission cannot be confined locally at 100 %, which also results in the high number of infections (Fig. 7). For example, when the local vaccination fraction in terms of degree-based, betweenness-based and strength-based control strategies reaches to 30 %, a high number of individuals (i.e., 25–45) in Fig. 7 are infected at a very low percentage (5–10 %) in Fig. 5. Those results suggest that containing disease outbreaks locally should be highly recommended, but the follow-up control strategies (e.g., vaccination) are needed if the local control strategies are not successful.

### Vaccination strategies in terms of spatial–social extent of infection

**Fig. 8:**
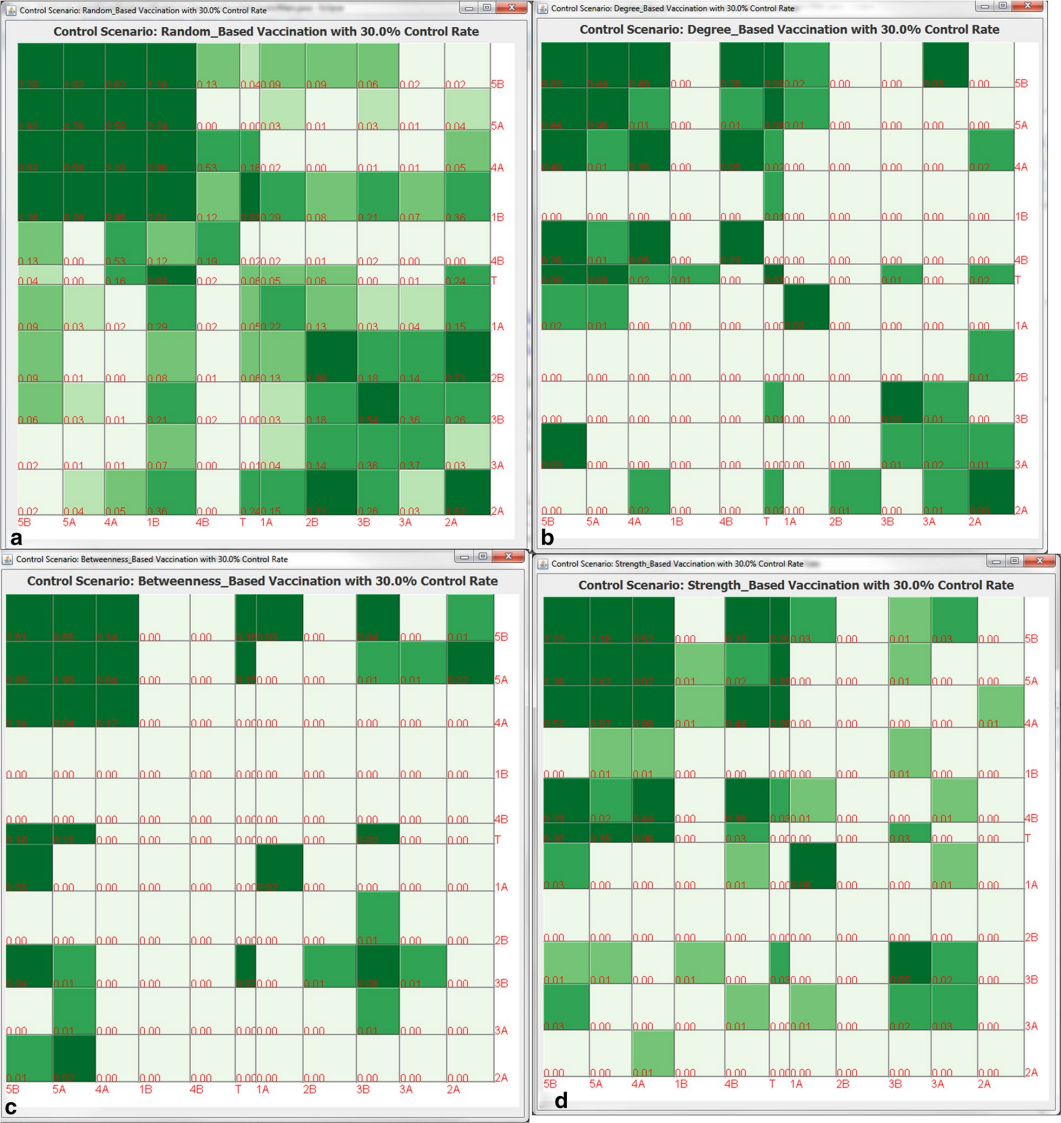
The spatial–social patterns of simulated vaccination strategies with 30 % vaccination rate with the average number of infections based on 10,000 runs after reordering the second day network, Friday, October 2nd 2009. **a** The random-based vaccination strategy, **b** the degree-based vaccination strategy, **c** betweenness-based vaccination strategy, and **d** the strength-based vaccination strategy

**Fig. 9:**
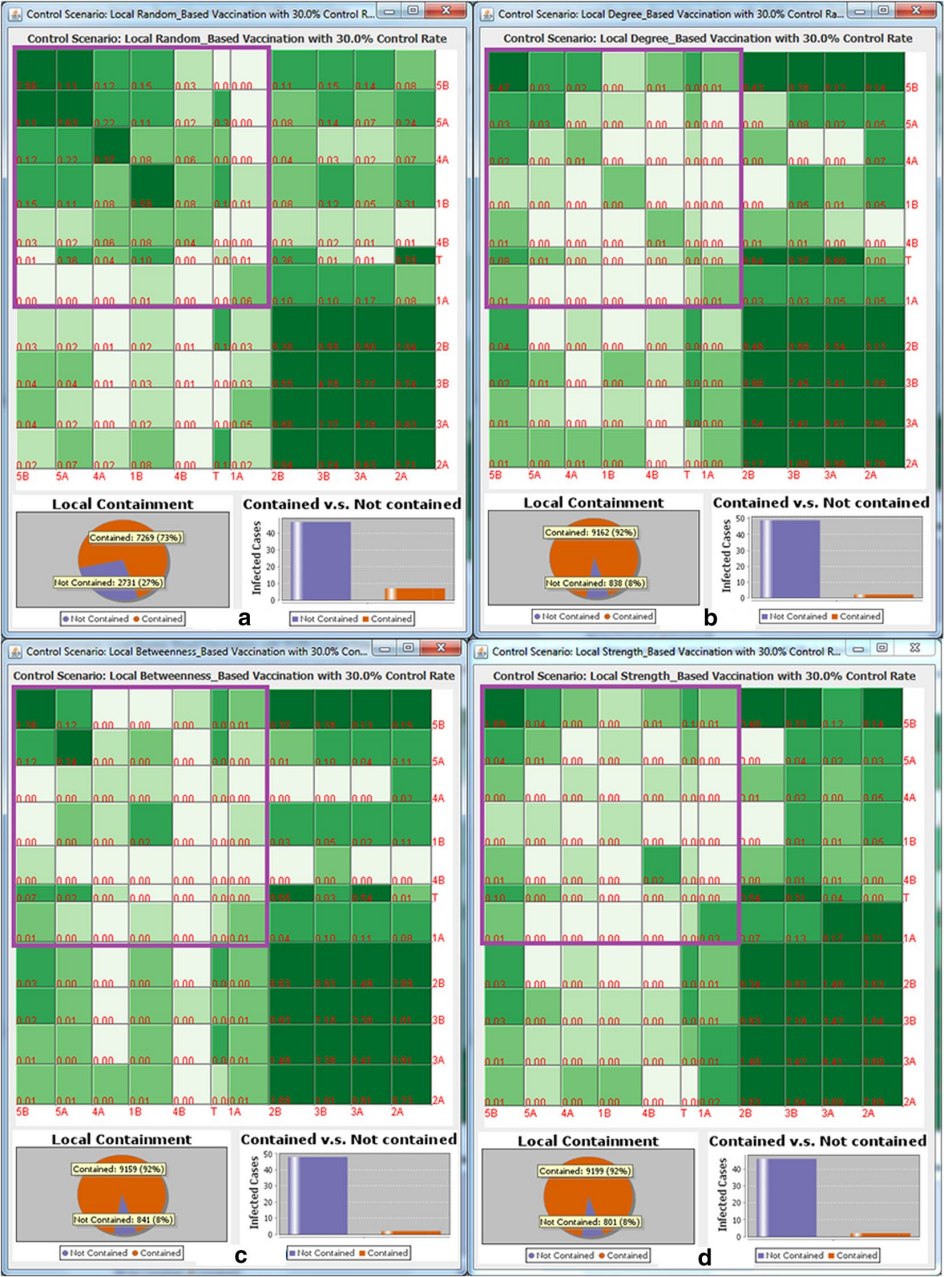
The spatial–social patterns of simulated vaccination strategies with 30 % vaccination rate based on the selected areas (*purple square*) after reordering with the average number of infections in each cell in the second day network, Friday, October 2nd 2009. The number of infections within the selected areas is achieved through calculating the sum of infected individuals on each of the 10,000 runs and then dividing by 10,000 runs. The average number of infections outside the selected areas is calculated through dividing by the number of local control failures, because the number of infections is zero when the local control is successful. **a** The random-based vaccination strategy, **b** the degree-based vaccination strategy, **c** betweenness-based vaccination strategy, and **d** strength-based vaccination strategy. The *pie chart in each figure* represents the number of local control success versus the number of local control failure with 10,000 simulation runs. The *bar chart in each figure* represents the average number of infections between the local control success and local control failure

From a spatial–social perspective, effective vaccination strategies with the selected areas are expected to confine the disease outbreak locally. This section only displays the simulation results with the second day, Friday, October 2nd 2009, because the 2 days’ results will generate the same conclusion as below. Figures 8 and 9 compare spatial–social extent of affected areas between the whole area and the selected area control scenarios with 30 % vaccination rate. The average number of infections based on the simulation results is displayed on each cell in each matrix. Within the selected areas in each matrix (in purple square), local control scenarios can produce a much lower number of infections (Fig. 9) compared to the whole area control scenarios (Fig. 8). This is because placing the same amount of vaccines within a smaller spatial–social extent of areas would cause a lower number of infections locally. However, outside the selected areas, there are a much larger number of infections (Fig. 9) compared to the whole area control scenarios (Fig. 8). Each pie chart in Fig. 9 shows the number of local control successes versus the number of local control failures with 10,000 simulation runs. Each bar chart in Fig. 9 represents the average number of infections between the local control success and local control failure. It shows that there is a high probability (see pie chart) to contain the disease outbreak locally with 30 % vaccination rate, but a larger disease outbreak would occur (see bar chart) if the local control fails at a low probability. Those results suggest that if the local control scenarios fail, new control strategies have to be implemented to avoid disease outbreaks outside the selected control areas. Therefore, the efficacy of vaccination strategies in terms of spatial–social extent of infection also suggests that local vaccination control would be a good control strategy. On the other hand, this strategy should be complemented with other control strategies if the local ones are not successful at a low likelihood.

### Vaccination strategies in terms of varying spatial–social scales

**Fig. 10:**
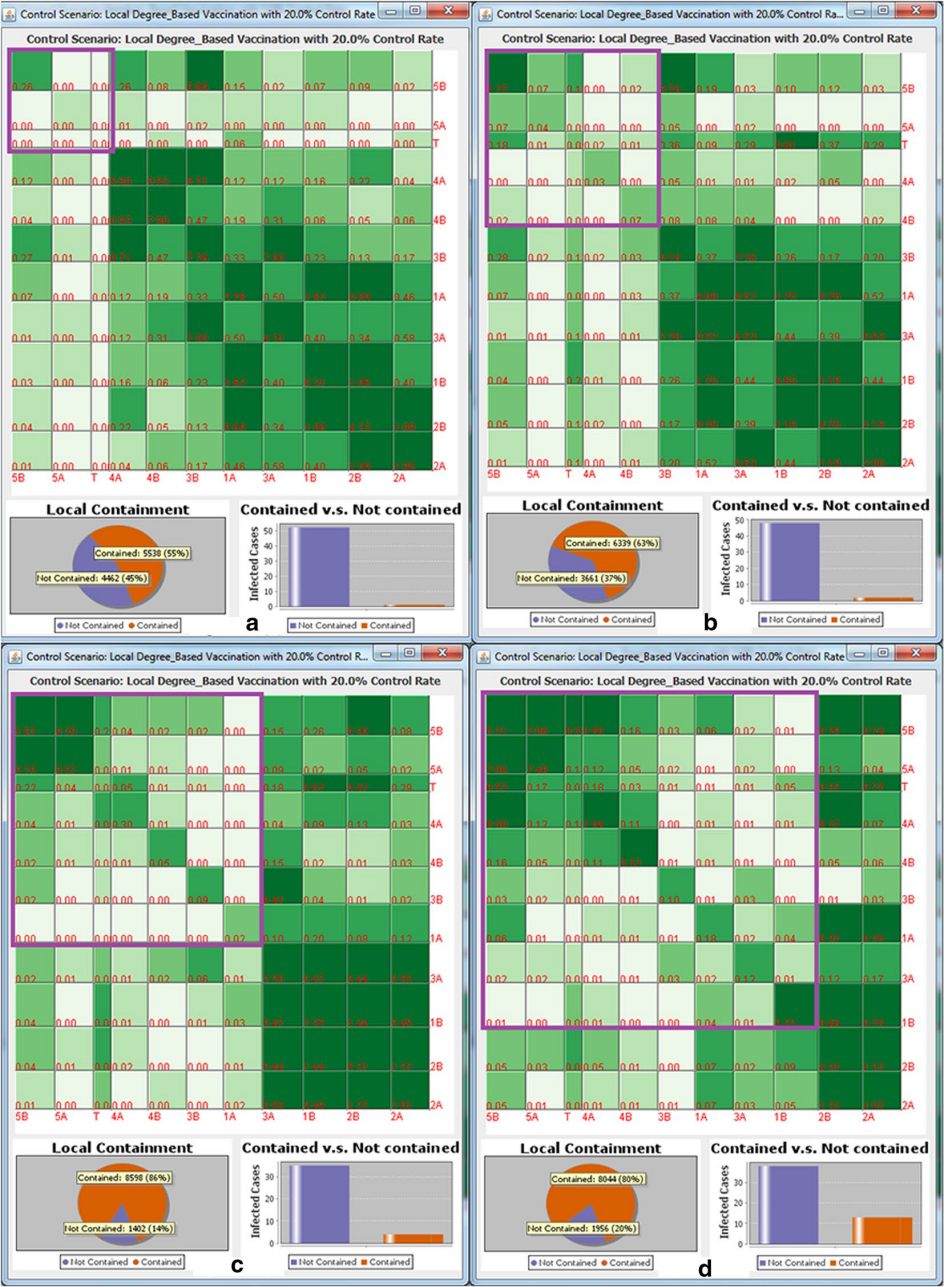
The spatial–social patterns of local degree based vaccination strategies with 20 % vaccination rate with varying spatial–social scales (in the *purple square*) after reordering with the average number of infections in each cell in the first day network, Thursday, October 1st 2009. The number of infections within the selected areas is achieved through calculating the sum of infected individuals on each of the 10,000 runs and then dividing by 10,000 runs. The average number of infections outside the selected areas is calculated through dividing by the number of local control. **a**–**d** Increasing spatial–social scales. The *pie chart in each figure* represents the number of local control success versus the number of local control failure with 10,000 simulation runs. The *bar chart in each figure* represents the average number of infections between the local control success and local control failure

This study further explores the impact of varying spatial–social scales on the success of local vaccination strategies. This section displays the simulation results with the local degree-based vaccination strategies on the first day, Thursday, October 1st 2009. Figure 10 compares simulation results with varying spatial–social scales (in the purple square) with a 20 % vaccination rate. Within the increasing spatial–social scales, the number of local control successes has a gradual increase from 55 % in Fig. 10a to 63 % in Fig. 10b to 86 % in Fig. 10c and decreases to 80 % in Fig. 10d. It implies that a proper spatial–social scale can help achieve the best control efficacy with a limited number of vaccinations. One explanation is that a certain number of vaccinations have its upper limit of susceptible population pool. The number of local control successes increases until it reaches the upper limit, whereas the number of local control successes decreases after it goes beyond the limit.

## Conclusions and implications

This research proposes a new framework in terms of effective disease control that starts from identifying geo-social interaction patterns, followed by designing effective control measures accordingly, and then evaluates the efficacy of different control measures. This framework is used to structure design of a new visual analytic tool: GS-EpiViz. This tool first identifies the geo-social interaction patterns applicable to the design/plan disease containment strategies before disease outbreak occurs, then implements the method and agent-based epidemic models into a visually interactive environment. With real world human interaction data as a case study, this research compares the efficacy of vaccination strategies between the spatial–social interaction patterns and the whole areas. The simulation results show that the control strategies based on spatial–social interaction patterns can lead to a significant reduction of epidemic size in terms of total number and spatial–social extent at a very high likelihood within the school environment. This research also gains new insights into how a proper spatial–social scale matters in terms of control efficacy with a limited number of vaccinations.

The success of vaccine strategies depends on early detection, efficient targeting, and prioritizing high risk individuals; this is essential because of the limited resources and time [47, 51]. This study provides valuable insights for designing effective control strategies that consider the geo-social interaction patterns and by doing so help meet the above challenges. Our approach demonstrates that the geo-social interaction patterns can help identify critical individuals, locations, and clusters of locations for disease control purposes. Geo-social interaction patterns should be used to implement control policies because simple distance threshold (e.g., 5 km) [32] cannot capture the most likely and complicated disease transmission processes. The varying spatial–social scales can help geographically and socially prioritize limited resources (e.g., vaccines) in time critical situations during an outbreak. After the first infections are reported, the varying spatial–social scales can help identify a proper scale for immediate actions. The distribution of available vaccines within the proper scale can also give an idea how likely it is that the infection can be confined within the scale. Based on the likelihood, policy makers can have a priority list according to limited resources and time to prepare for the situation when the infection cannot be confined within the scale.

Though the real, high-resolution human interaction data analysed here provides a proof of concept to study the impact of geo-social mixing patterns and scales on infectious disease control, three major limitations of this research are important to mention. First, the data used in this research measure the frequency of spatial proximity between individuals, but they do not include spatial topology information (e.g., relative position of the classrooms). Such spatial information has mostly been used to build human interaction network such as assigning individuals to home or workplace according to their relative positions [36, 52]. After the human interaction network is built, infectious disease simulation is based on the network topology rather than spatial topology. Thus, the agent-based simulation processes based on our data are similar as other models with spatial topology information. Second, though vaccination strategies we discussed in this research are novel, we only tested them with one specific dataset corresponding to one particular school. High resolution data collected from different schools or different countries [7] can capture varying human interaction patterns within the classes and among different classes. Lastly, though the high resolution data can capture the real human interaction patterns for epidemic analysis research purpose, its limitation comes from relatively small network size.

The above limitations illustrate several future research directions in terms of GS-EpiViz development with more complex human interaction data. First, insights could be achieved by comparing effectiveness of the proposed strategies using such high-resolution data describing the real spatial–social interaction patterns from other schools or countries. Second, It would represent an important step to apply the proposed strategies to human interaction data at a larger scale (e.g., urban). In reality, each classroom in this study can be viewed as one geographical location (e.g., workplace, home) at larger spatial scales (e.g., cities), whereas human movement among different classrooms can be viewed as spatial interactions among geographical locations. Gao and Bian [53] found that human interaction network within a metropolitan community is spatially clustered. Thus, the effectiveness of our vaccination strategies based on the school data with the high density of CPIs implies that those strategies are very likely to be effective to control disease spread with larger scale spatial–social network data with a large number of individuals move within and between communities on a daily basis such as urban areas [22]. We expect that the strategies would be more effective to control infectious disease transmissions with larger scale spatial–social network with weak connections between sparse population distributions such as rural areas [22]. In terms of network size, the space complexity in GS-EpiViz is O(m + n + k^2^), in which m represents the total number of edges, n represents the number of nodes, and k represents the number of communities. Then it would not be a problem for GS-EpiViz to deal with large data sets (up to 1 GB). For example, a network with 1,000,000 nodes and 5,000,000 edges and 1000 communities requires approximately 1 gigabytes of internal memory for handling the data all at once.

In summary, infectious disease transmission is determined by the mixed interactions of the social and spatial relationships among individuals [17, 54]. Either relationship can play an important role in exploring proper vaccination strategies. Social or spatial relationships, respectively, have received substantial attention, while the mixed interactions of the social and spatial relationships have often been under-studied [22]. To our knowledge, this research is the first attempt to evaluate the efficacy of control scenarios when considering geo-social interaction patterns and to bring the concept of scale into the design of control scenarios. GS-EpiViz facilitates the vaccination strategy evaluation with local spatial–social interaction patterns and varying spatial–social scales. The results provide insights into community-based planning within school environment and potentially at larger spatial scale (e.g., urban, rural) for controlling emerging air-borne infectious diseases.
